# β_3_ phosphorylation of platelet α_IIb_β_3_ is crucial for stability of arterial thrombus and microparticle formation in vivo

**DOI:** 10.1186/s12959-017-0145-1

**Published:** 2017-08-30

**Authors:** Weiyi Feng, Manojkumar Valiyaveettil, Tejasvi Dudiki, Ganapati H. Mahabeleshwar, Patrick Andre, Eugene A. Podrez, Tatiana V. Byzova

**Affiliations:** 1 Department of Molecular Cardiology, The Cleveland Clinic Foundation, Cleveland, 44195 OH USA; 2 Plaint Therapeutics, Redwood City, CA USA; 3 The First Affiliated Hospital, School of Medicine, Xi’an Jiaotong University, Xi’an, Shaanxi 710061 China; 4 US Army Medical Materiel Development Activity, 1430 Veterans Drive, Fort Detrick, Frederick, MD 21702 USA

**Keywords:** β_3_ integrin phosphorylation, Outside-in integrin signaling, Microparticles, Arterial thrombosis

## Abstract

**Background:**

It is well accepted that functional activity of platelet integrin α_IIb_β_3_ is crucial for hemostasis and thrombosis. The β_3_ subunit of the complex undergoes tyrosine phosphorylation shown to be critical for outside-in integrin signaling and platelet clot retraction ex vivo. However, the role of this important signaling event in other aspects of prothrombotic platelet function is unknown.

**Method:**

Here, we assess the role of β_3_ tyrosine phosphorylation in platelet function regulation with a knock-in mouse strain, where two β_3_ cytoplasmic tyrosines are mutated to phenylalanine (DiYF). We employed platelet transfusion technique and intravital microscopy for observing the cellular events involved in specific steps of thrombus growth to investigate in detail the role of β_3_ tyrosine phosphorylation in arterial thrombosis in vivo*.*

**Results:**

Upon injury, DiYF mice exhibited delayed arterial occlusion and unstable thrombus formation. The mean thrombus volume in DiYF mice formed on collagen was only 50% of that in WT. This effect was attributed to DiYF platelets but not to other blood cells and endothelium, which also carry these mutations. Transfusion of isolated DiYF but not WT platelets into irradiated WT mice resulted in reversal of the thrombotic phenotype and significantly prolonged blood vessel occlusion times. DiYF platelets exhibited reduced adhesion to collagen under in vitro shear conditions compared to WT platelets. Decreased platelet microparticle release after activation, both in vitro and in vivo, were observed in DiYF mice compared to WT mice.

**Conclusion:**

β_3_ tyrosine phosphorylation of platelet α_IIb_β_3_ regulates both platelet pro-thrombotic activity and the formation of a stable platelet thrombus, as well as arterial microparticle release.

## Background

Platelet activation and aggregation control physiological defense mechanisms such as cessation of bleeding after injury, and also underlie the pathophysiology of ischemic disorders, stroke and myocardial infarction [1, 2]. Several signaling pathways trigger platelet activation in vivo, but the common result is the activation of the major platelet surface glycoprotein, integrin α_IIb_β_3_ [1, 3–5]. On platelets, integrin α_IIb_β_3_ is a key receptor whose activity is rapidly induced upon agonist stimulation (ADP, thrombin, etc.), resulting in binding of its major physiological ligand, plasma fibrinogen, and subsequent platelet aggregation [4]. The blockade of α_IIb_β_3_ with antibodies, peptides, peptidomimetics or small compounds results in reduced thrombotic activity and prolonged occlusion times [6, 7]. Animals deficient for β_3_ integrin are characterized by prolonged bleeding times, gastrointestinal hemorrhage, and abnormal platelet aggregation and clot retraction [8, 9].

The α_IIb_β_3_ cytoplasmic domains serve as docking sites for numerous intracellular adaptors, signaling molecules and cytoskeletal proteins. These interactions are crucial for both inside-out and outside-in signaling processes [10, 11]. The β_3_ subunit of α_IIb_β_3_ contains two cytoplasmic tyrosine residues, Tyr747 and Tyr759. Tyrosine phosphorylation of the β_3_ was shown to occur during platelet aggregation as a result of fibrinogen binding to the receptor; it is considered to be a process triggered by outside-in integrin signaling [12–14]. Phosphorylation of β_3_ was shown to be crucial for recruitment of several adaptor molecules, including Shc, Grb and cytoskeletal myosin, a protein crucial for retraction of the fibrin clot by platelets [4, 15].

The direct role of β_3_ tyrosine phosphorylation in the regulation of platelet functions was tested using the knock-in mouse strain DiYF, where both tyrosines were mutated to phenylalanine. The resultant mutant α_IIb_β_3_ is unable to undergo phosphorylation and interact with adaptor molecules, which severely affects platelet functions. Aggregation of DiYF platelets was reported to be reversible with defective clot retraction responses, resulting in the tendency of DiYF mice to re-bleed in the standard tail bleeding assay [9]. Importantly, the β_3_ subunit in complex with α_V_ is present on several populations of blood cells, including monocytes and T lymphocytes, and also on vascular endothelial cells [16]. Besides platelets, the DiYF mutation affects functions of other cells known to serve as important contributing factors in thrombus formation [17]. Our recent studies showed that the DiYF mutation results in a series of abnormalities in endothelial cell adhesion, spreading, and migration [18]. Thus, endothelial defects in DiYF mice might be critical for thrombus initiation in vivo. Additionally, it is known that heterotypic interactions between platelets and blood mononuclear cells serve as another important mechanism regulating thrombus progression [19].

Accordingly, the aim of this study was to comprehensively analyze the role of β_3_ tyrosine phosphorylation within an in vivo model of arterial thrombosis and to dissect the role of the platelet-specific component. For the latter objective, we have developed a new in vivo model involving platelet transfusion which allows analysis of DiYF mutation exclusively on platelets.

## Methods

### Mice

Eight- to 12-week-old, sex- and age-matched wild type (WT, C57BL/6) or DiYF mice were used in this study. Seven generations of back crossed GFP^+/+^-C57BL/6 mice were purchased from The Jackson Laboratory (Bar Harbor, ME). GFP^+/+^-DiYF mice were bred from DiYF and GFP heterozygous mice. All animal procedures were performed in accordance with an approved institutional protocol according to the guidelines of the Institutional Animal Care and Use Committee of the Cleveland Clinic.

### Intravital microscopy

Blood was collected from the abdominal vein of WT or DiYF mice and treated with 1/10 vol of acid-citrate-dextrose anticoagulent containing 1 μg/ml prostaglandin E_1_ (Sigma-Aldrich, St. Louis, MO). WT or DiYF platelets were obtained and labeled with calcein green (Invitrogen, Carlsbad, CA), and then infused into the respective WT or DiYF mice (4-5 × 10^6^ /g) via tail vein as previously described [20]. Mice were anesthetized and placed on a 37 °C warm platform. The carotid artery was exposed and visualized using a Leica DMLFS fixed stage microscope. Images were recorded with a high speed color cooled digital camera (Q-imaging Retiga Exi Fast 1394) with Streampix^R^ high speed acquisition software. Leica water immersion objectives at 10× −63× were used. To initiate thrombosis, a patch (1.5 × 1.5 mm) of filter paper saturated with 10% FeCl_3_ solution was placed on the carotid artery for 2 min. The blood flow and platelet vessel wall interactions taking place in the carotid artery were monitored continuously for 60 min after vessel wall injury, or until full occlusion occurred and lasted for more than 30 s.

### Perfusion chamber experiments

The thrombosis phenotype of WT and DiYF mice was evaluated in the murine ex vivo perfusion chamber protocol exposing fibrillar collagen to non-anticoagulated samples of blood under arterial shear rates as previously described [21]. Briefly, non-anticoagulated blood was collected from the vena cava of anesthetized mice and perfused for 2.5 min through human type III collagen-coated capillary chambers. Capillary chambers with a diameter of 345 μm were used to establish a shear rate of 871/s (flow rate of 212 μl/min). Mean thrombus volume (μm^3^/μm^2^) was quantified on semi-thin cross section and by mean grey level measurements at 5 mm from the proximal part of the capillary using Simple PCI software.

### Irradiation and platelet transfusion model

WT mice were depleted of blood cells by sublethal γ–irradiation (6 Gy) then were subdivided into two groups 14 days later. 5 × 10^8^ WT or DiYF platelets (10% of the platelets were labeled fluorescently with calcein green) in 0.3 ml Tyrode’s buffer pH 7.4 (composition [mm]: NaCl 134, NaHCO_3_ 12, KCl 2.9, MgCl_2_ 1, CaCl_2_ 2, HEPES 5, supplemented with 5 mm glucose and BSA) were infused into each irradiated mouse through the tail vein in their respective WT and DiYF groups. The in vivo thrombosis procedure was performed and thrombus formation was observed in these mice. Carotid artery occlusion was induced as described above for 3 min using filter paper saturated with 15% FeCl_3_.

### Tail-bleeding measurements

WT mice were infused with 5 × 10^8^ WT or DiYF platelets 2 weeks after irradiation as described above. Platelet suspension buffer was injected for the control mice. Two hours after infusion, mice were anaesthetized and placed on a 37 °C warm platform before having 2 mm of the tip of their tails cut with a sharp scalpel. The time required for the flow of blood to cease was recorded.

### Clot retraction experiments

Platelet rich plasma (PRP) was obtained from blood of irradiated and WT or DiYF platelet transfused WT mice (WT-WT(platelet) or WT-DiYF(platelet) mice, respectively). Platelet concentration in PRP was adjusted to 2 × 10^5^/μl with PBS. Clot retraction was measured by mixing the following in an aggregometer tube: 100 μl of PRP, 160 μl of clot retraction buffer (PBS solution containing 1 mM CaCl_2_ and 1 mM MgCl_2_), 50 μl of working solution of thrombin (clot retraction buffer with 8 U/ml thrombin). For visualization, 5 μl of WT erythrocytes were added. A glass rod was placed upright in the test tube which was incubated at 37 °C. Clot formation was checked and the volume of remaining solution was measured after 30 min incubation.

### Flow cytometry analysis of platelet α_IIb_β_3_ activation

Gel-filtered platelets (1.0 × 10^6^) from WT-WT(platelet) or WT-DiYF(platelet) mice were resuspended in Tyrode’s buffer containing 2 mM Ca^2+^ and 1 mM Mg^2+^. Platelets were stimulated either with ADP (10 or 2 μM) or thrombin (0.1 or 0.05 U/ml) for 5 min at room temperature. The activated platelets were incubated with PE-conjugated anti-α_IIb_β_3_ antibody (Emfret Analytics, Germany) for 15 min. Data for 20,000 positive cells were acquired using a FACSCalibur instrument (BD Biosciences, San Jose, CA).

### Platelet aggregation

Gel filtered platelets were prepared as described above. Platelet aggregation was monitored using a Lumi-Aggregometer type 500 VS (Chrono-log Corporation, Havertown, PA). Thrombin (0.1, 0.075 and 0.05 U/ml) and collagen (5, 2.5 and 1 μg/ml) were used as agonists.

### Platelet adhesion

5 × 10^7^ GFP-transgenic WT or DiYF platelets (in 250 μl Tyrode’s/BSA buffer) were added to collagen coated wells of a 6–Well culture plate at 37 °C in an orbital shaker at a maximum speed of either 380 cm/min (36 RPM) or 640 cm/min (60 RPM) and incubated for 1 h. The wells were washed with Tyrode’s buffer and the images of bound platelets were acquired by fluorescence microscopy. Image quantification was performed using ImagePro software.

### Preparation and flow cytometry analysis of microparticles from platelets

4 × 10^7^ WT or DiYF platelets in suspension were activated by either thrombin (0.5 U/ml) or 200 nM phorbol-12-myristate-13-acetate (PMA, Calbiochem, San Diego, CA) for 15 min at 37 °C. Samples were then centrifuged at 12,000×g for 10 min at 22 °C to remove the platelets. The microparticle-enriched supernatant was harvested and stained with FITC-labeled annexin V (BD Biosciences, San Diego, CA) for 15 min in the dark at room temperature. The positive microparticle population was analyzed using a FACSCalibur instrument.

For in vivo experiments, a modified ferric chloride model of arterial thrombosis was used [22]. Briefly, WT and DiYF mice were anesthetized with ketamine/xylazine, a midline cervical incision was made and the mesentery was exposed by blunt dissection at 37 °C. 0.2 ml of 2% FeCl_3_ solution in saline was applied to the surface of the vessels in the mesentery. After 5 min, blood was collected from the abdominal vein, labeled with FITC-conjugated annexin V and the positive microparticles in whole blood were measured by flow cytometry.

### Statistical analysis

Results are reported as the mean ± SEM. Statistical significance was assessed by unpaired Student’s t test. The non-parametric log-rank test was used to analyze occlusion and bleeding times. *P* values <0.05 were considered significant.

## Results

### Unstable thrombi and prolonged occlusion time in DiYF mice

**Fig. 1:**
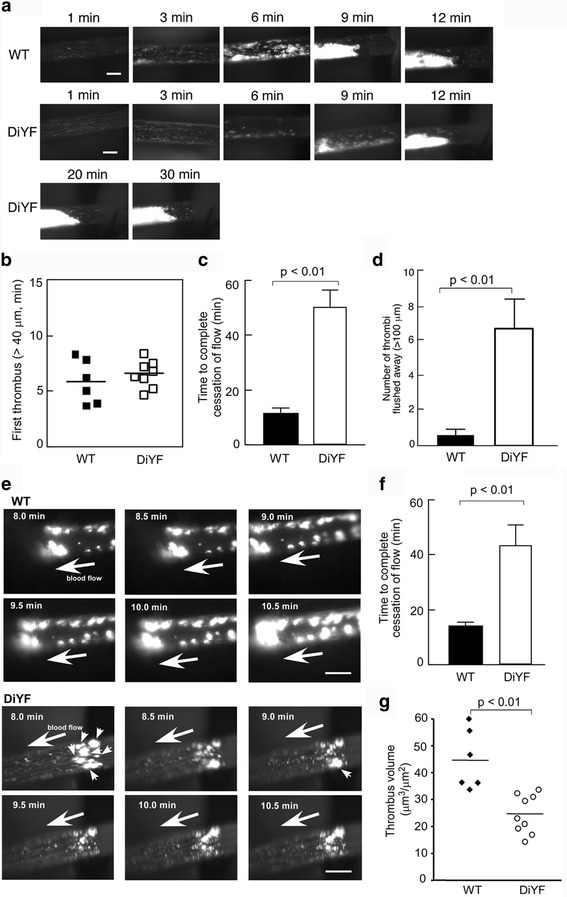
Delayed thrombosis in DiYF mice. **a** Characteristics of thrombus growth in WT and DiYF mice in the carotid artery after injury. Bars = 500 μm. **b** No significant difference in the time to first thrombus formation (>40 μm) between WT (*n* = 6) and DiYF (*n* = 8) mice. **c** Delayed thrombus formation in DiYF mice (*n* = 8) compared to WT mice (*n* = 6). **d** Numbers of thrombi (>100 μm) removed by blood flow in WT and DiYF mice 10 min after carotid injury. **e** Characteristics of thrombi removed by blood flow in DiYF mice. **→** show blood flow, → show detached/flushed thrombi. Bars = 500 μm. **f** Delayed thrombus formation in GFP-transgenic DiYF mice (*n* = 6) compared to GFP-transgenic WT mice (*n* = 6). **g** Decreased thrombus volumes from DiYF blood formed on collagen in capillary chambers compared to their WT counterpart. Error bars represent SEM

As Fig. 1a shows, blood vessel injury resulted in the rapid attachment of platelets to the damaged site with the subsequent formation of platelet microaggregates. Adherent platelets and small aggregates of platelets appeared approximately 3-8 min after injury, both in WT and DiYF groups (Fig. 1b). Video analysis revealed that the formation of platelet microaggregates in DiYF mice was slightly but not significantly delayed in the initial stages of thrombus formation. The average times for the first thrombus ≥40 μm size formed in the carotid were 5.8 min in WT mice and 6.5 min in DiYF mice (Fig. 1b). Compared to WT, the subsequent growth rate of the thrombus appeared to be generally normal in DiYF mice. In WT mice the platelet thrombus occluded the blood vessel causing the complete cessation of blood flow around 12 min after injury (Fig. 1a and c). However, in DiYF mice, despite the presence of a thrombus, blood flow still continued for a prolonged period of time. As shown in Fig. 1a and c, the carotid arteries of DiYF mice remained open after 12 min time point with the continuous high shear, whereas the blood flow was completely disrupted in the carotid arteries of WT mice. On average, the time to complete cessation of blood flow was prolonged in DiYF mice by ~5 fold as compared to WT mice (Fig. 1c). In most cases with the DiYF mice (6 out of 8), the experiment was stopped at 60 min, even though complete occlusion of the carotid artery was not achieved. Video analysis showed that as thrombus formation progressed, parts of or even entire thrombi formed in DiYF mice were loosely packed. These loosely packed thrombi in DiYF mice were easily detached by flowing blood after they had grown larger, up to 100-200 μm. ImagePro analysis of the video revealed that thrombi formed in DiYF mice were ~50% less stable than that formed in WT mice. This defect resulted in the delayed visual accumulation of aggregated platelets at the site of injury (Fig. 1a) and dramatically prolonged occlusion times in DiYF mice (Fig. 1c). In addition, approximately 7 thrombi (≥100 μm size) were washed away in each DiYF mouse during the 10 min after the first thrombus appeared (Fig. 1d and e). Meanwhile, no thrombi were washed away in most of the WT mice (Fig. 1d and e). Thus, it appears that reversible platelet aggregation previously observed in DiYF mice causes the formation of a fragile and unstable thrombus, which, in turn, is responsible for substantially delayed arterial occlusion upon injury.

To make sure that calcein green labeling was not affecting platelet function, we repeated the same in vivo thrombus experiments by using WT and DiYF GFP-transgenic mouse platelets. The microthrombi formed in DiYF mice were still looser and less stable than in WT. Blood flow was occluded around 15 min in WT mice, but only three out of six DiYF mice reached occlusion before 60 min after injury, confirming the previous observations and the functional activity of platelets (Fig. 1f).

To further investigate the initial stages of thrombosis in DiYF mice, we analyzed thrombus formation on collagen under high shear conditions. The results showed that DiYF thrombi formed on the collagen surface were significantly smaller than their WT counterparts (Fig. 1g), suggesting that impaired β_3_ phosphorylation also affects the process of platelet adhesion to and thrombus formation on collagen substrate.

### Delayed thrombus formation in WT mice with DiYF platelets in vivo

**Fig. 2:**
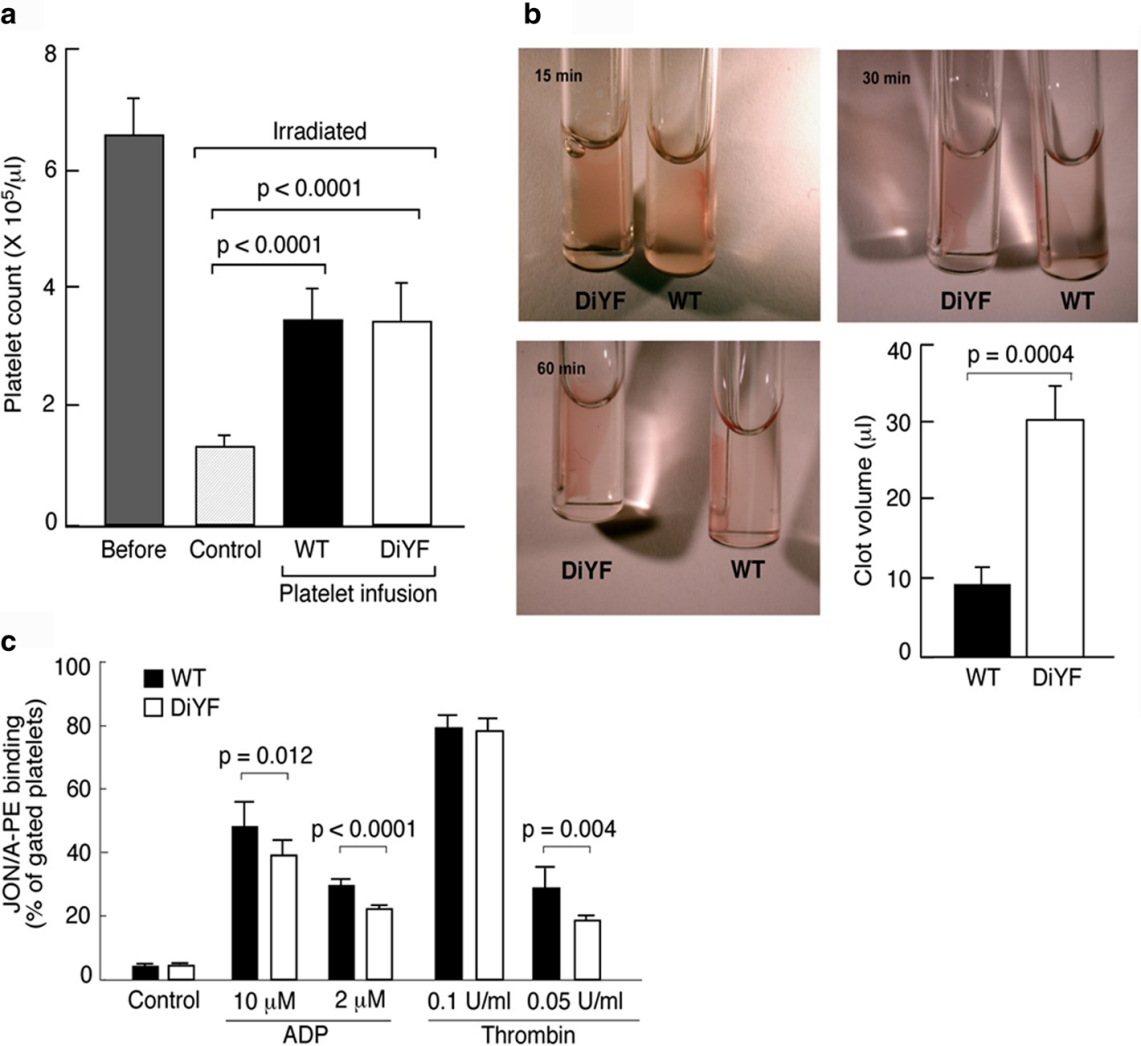
Defective clot retraction and α_IIb_β_3_ activation upon transfusion of DiYF but not WT platelets. **a** Platelet counts before irradiation (*n* = 12), after irradiation (control, *n* = 12) and after platelet transfusions (both *n* = 8). **b** Defective platelet retraction function in WT mice with DiYF platelets; Characteristics of clot retraction after 15, 30 and 60 min in transfused WT and DiYF PRP samples. Quantification of significantly increased clot volumes in transfused DiYF platelets compared to WT platelets is shown as a graph in the bottom right panel (mean ± SEM from five independent experiments). **c** Defective platelet α_IIb_β_3_ activation in WT mice with DiYF platelets (mean ± SEM from three independent experiments)

**Fig.3:**
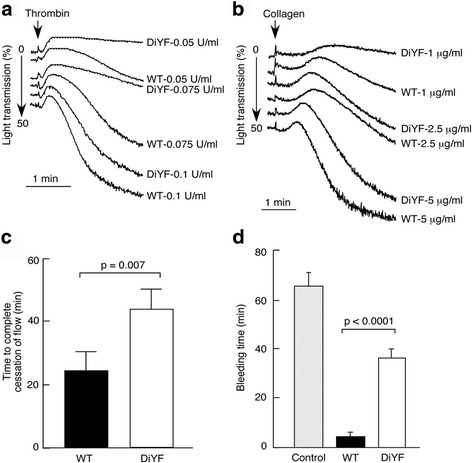
**a** and **b** Aggregation assays of platelet function in WT mice with WT or DiYF platelets (representative curves from three independent experiments). **c** Delayed thrombus formation in irradiated mice transfused with DiYF but not WT platelets (mean ± SEM from nine independent experiments). **d** Prolonged bleeding time in WT mice with DiYF platelets compared to their WT counterparts (mean ± SEM, *n* = 5)

As shown in Fig. 2a, irradiation caused at least a 5-fold reduction in platelet counts while transfusion of either WT or DiYF platelets showed a 2.5-fold increase. Clot retraction by the platelets from WT-DiYF(platelet) mice was significantly delayed compared to their WT counterparts (Fig. 2b), indicating defective platelet retraction, consistent with a previous report on WT and DiYF platelets [9]. Moreover, upon stimulation with ADP and thrombin, α_IIb_β_3_ activation on the surface of platelets from WT-DiYF(platelet) mice was significantly impaired compared to the WT-WT(platelet) group (Fig. 2c; the difference was notable at lower concentrations of agonist). In aggregation assays, platelets from the WT-DiYF(platelet) group failed to aggregate upon stimulation with either 0.05 U/ml of thrombin or 1 μg/ml of collagen, compared to WT-WT(platelet) mice (Fig. 3a and b). At higher concentrations of agonist (0.1 U/ml thrombin or 5 μg/ml collagen), there was no significant difference in the aggregation curves (Fig. 3a and b), similar to previous observations [9]. Thus, transfusion of irradiated WT mice with DiYF platelets produced a platelet-specific DiYF phenotype.

Next, WT-WT(platelet) and WT-DiYF(platelet) mice were tested with the carotid injury model. Around 30% of thrombi formed in WT-DiYF(platelet) mice were unstable and repeatedly detached, in contrast to the WT-WT(platelet) group where thrombi were consistently stable. This phenomenon occurred in most WT-DiYF(platelet) mice but not in WT-WT(platelet) mice. Accordingly, the time to complete cessation of blood flow in WT-DiYF(platelet) mice was significantly longer than in the WT-WT(platelet) group (Fig. 3c).

Measurements of tail bleeding times in mice transfused with WT and DiYF platelets revealed that WT-DiYF(platelet) mice bled 5 times longer than WT-WT(platelet) mice (Fig. 3d). Interestingly, it was previously observed that while DiYF mice exhibited a pronounced tendency to re-bleed after transient hemostasis, the bleeding times were not substantially prolonged [9]. It is possible that in these animals with overall low blood cell counts, the typical tail cut model presents a greater hemostatic challenge and reveals a relatively subtle phenotype caused by the DiYF mutation in platelets.

Thus, it appears that the hemostatic defect observed in DiYF mice is primarily linked to the abnormal function of β_3_ integrin on platelets rather than on other cell types. Moreover, defective phosphorylation of β_3_ in platelets, which impairs outside-in integrin signaling, affects thrombus structure and stability in vivo.

### Reduced adhesion of DiYF platelets to type I collagen

**Fig. 4:**
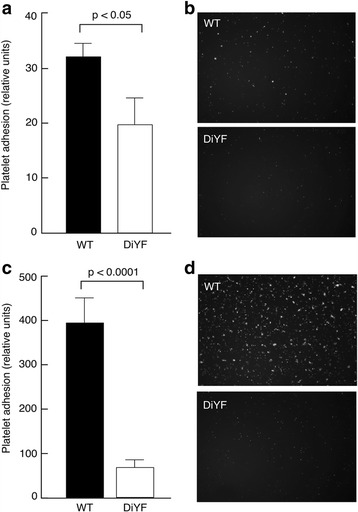
Reduced DiYF platelet adhesion to collagen type I compared to WT platelets. Platelet adhesion and accumulation on collagen at shaker rates of 380 cm/min (**a**, **b**) and 640 cm/min (**c**, **d**) are shown (mean ± SEM from three independent experiments)

Quantitative analysis of platelet adhesion to collagen at a lower velocity (380 cm/min) revealed that the DiYF mutation caused a 40% decrease in the number of adherent platelets compared to WT (Fig. 4a and b). At the same time, the difference in adhesion of WT and DiYF platelets was much more dramatic at higher velocity (640 cm/min; Fig. 4c and d). While WT platelets firmly adhered to collagen coated plates and formed small aggregates, attachment of DiYF platelets was reduced by at least 5-fold. Thus, the defects in firm adhesion of DiYF platelets can only be revealed under conditions of higher velocity, which, in turn, should primarily affect thrombosis at the arterial side of circulation.

### Critical role of β_3_ tyrosine phosphorylation in platelet microparticle release

**Fig. 5:**
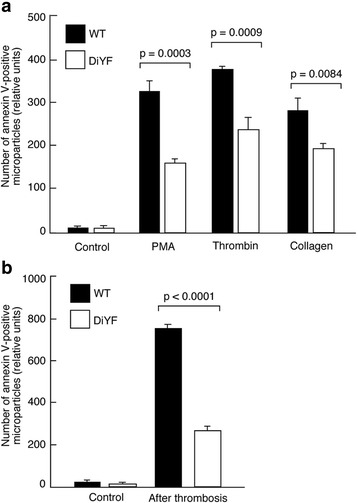
Defective microparticle formation by DiYF platelets in vitro and in vivo. **a** Decreased annexin V-positive microparticles generated by DiYF platelets compared to WT platelets. **b** Reduced microparticles originating from platelets after thrombus formation in DiYF mice compared to their WT counterparts. Mean ± SEM from three independent experiments

In view of increasing evidence that platelet microparticles are particularly important for in vivo thrombosis [23–27], we assessed whether impaired β_3_ phosphorylation affected microparticle release by activated platelets in vitro and in vivo. Platelet stimulation with PMA, thrombin or collagen resulted in a substantial release of microparticles and DiYF platelets shed approximately 50% fewer microparticles compared to WT platelets with all the agonists tested (Fig. 5a).

Importantly, reduced circulating microparticle levels were observed in DiYF mice compared to WT when multiple thrombosis processes were triggered by FeCl_3_in mesenteric blood vessels. The presence of annexin V-positive microparticles in the blood of DiYF mice was decreased by 3-fold compared to WT (Fig. 5b). Thus, it appears that β_3_ integrin tyrosine phosphorylation is critical for microparticle release upon platelet activation both in vivo and in vitro.

## Discussion

Using knock-in DiYF mice we assessed the role of β_3_integrin tyrosine phosphorylation in the regulation of arterial thrombosis in vivo with a particular focus on platelet-specific effects. Our major findings are: 1) the thrombus formed in the DiYF mouse is unstable, thus is easily detached by blood flow resulting in delayed occlusion of injured arteries*.* 2) Delayed thrombosis is due to impaired β_3_ phosphorylation on platelets but not on other cells expressing β_3_ integrin. 3) Defective β_3_phosphorylation results in impaired adhesion of DiYF platelets to collagen under shear conditions. 4) β_3_phosphorylation is crucial for microparticle release by activated platelets. Platelet microparticle levels were reduced in DiYF mice compared to WT in vivo and in vitro.

**Fig. 6:**
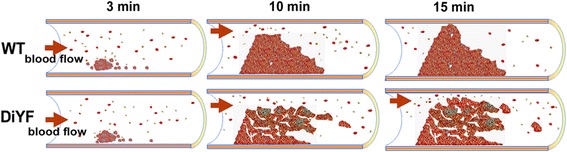
Model illustrating the instability of thrombus in DiYF mice. The growth of in vivo thrombus formation is depicted in the form of a cartoon in both WT and DiYF mice at different time intervals. Even though the thrombus growth rate is comparable for both WT and DiYF mice, the DiYF mice thrombi are loose and fragile compared to WT thrombi. The arrow indicates blood flow direction

We employed intravital microscopy, a powerful tool for observing the cellular events involved in specific steps of thrombus growth [8], to investigate in detail the role of β_3_ tyrosine phosphorylation in arterial thrombosis in vivo. We show that the time required for complete cessation of blood flow was substantially prolonged in DiYF knock-in mice. This phenomenon was not due to delayed or impaired progression of thrombus growth, but due to loose platelet-platelet bonds and an overall reduced stability of platelet aggregates (Fig. 6). Thrombi formed in DiYF but not in WT mice were easily detached and washed away by blood flow in injured arteries. In vivo, only a modest delay in the early stages of thrombus formation was observed in DiYF mice vs WT. However, more detailed ex vivo analysis revealed impaired platelet adhesion and thrombus formation by DiYF platelets on collagen under shear conditions. This defect might be crucial during initiation of thrombotic events when collagen matrix is a strong contributing factor. Although platelet attachment to collagen under flow is mediated by GPVI and α_2_β_1_, α_IIb_β_3_ serves as a crucial mediator of platelet-platelet interactions and the formation of microaggregates [28]. Activation of α_IIb_β_3_ was reported to serve as a prerequisite for α_2_β_1_ activation and interaction with collagen [29]. Interestingly, high shear conditions were able to reveal substantial defects in adhesion of phosphorylation-defective DiYF platelets to collagen, indicating that β_3_ phosphorylation is crucial for this process. It is possible that impaired α_IIb_β_3_ outside-in signaling might affect activation of collagen receptor α_2_β_1_.

Integrin β_3_ (in combination with αv) is expressed on endothelial cells as well as on circulating blood cells such as monocytes, lymphocytes and neutrophils. These cellular components are known to be critical for thrombus formation in vivo [30–33]. For obvious reasons, DiYF bone marrow transplantation into WT mice was only partially helpful to observe platelet functions in vivo, since it would affect all circulating blood cells. Accordingly, we developed a new platelet transfusion technique which allows the assessment of platelet-specific effects rather than broad platelet-leukocyte-vascular phenotype features of mice with modified function of β_3_ integrin. Using this model we demonstrate in DiYF mice that the phenomenon of unstable thrombi is caused by defective platelet function, not endothelial cells or leukocytes. As we reported previously, endothelial cells of DiYF mice display a series of abnormalities including impaired integrin-dependent adhesion and spreading [18, 34]. Although these functions are crucial for angiogenesis, they do not substantially contribute to arterial thrombosis, which appears to be solely dependent on platelet β_3_ integrin and its phosphorylation.

Another mechanism responsible for the overall delay of in vivo thrombosis in DiYF mice is the defective shedding of annexin V-positive microparticles by DiYF platelets. Microparticle formation accompanies in vivo platelet activation and greatly promotes the process of thrombin generation, further accelerating platelet activation and aggregation [35–38]. Our finding is further supported by an observation that platelets from Glanzmann thrombasthenia patients also have decreased microparticle release when stimulated with various agonists, as compared with normal human platelets [39], thereby solidifying the role for α_IIb_β_3_ signaling in microparticle generation. Clinical studies demonstrated that elevated platelet microparticles play an important role in the pathogenesis of the prothrombotic state in patients suffering from a number of diseases such as systemic lupus erythematosus. Patients with lupus have a significantly increased risk for thrombosis which affects both venous and arterial vessels [23, 40]. Thus, platelet microparticles, which are highly active in thrombin generation and fibrin clot formation processes as well as in amplification of platelet aggregation, appear to contribute to thrombosis in certain clinical settings [41]. Here we provide evidence that platelet β_3_ phosphorylation and outside-in signaling promotes the release of microparticles upon platelet activation, which in turn, might accelerate the later stages of thrombus progression. Thus, not only reversible platelet aggregation in DiYF mice, but also impaired platelet-collagen interactions under shear and reduced shedding of microparticles are factors contributing to the formation of fragile and unstable thrombi in DiYF mice, which in turn, is responsible for substantially delayed arterial occlusion upon injury.

Our study highlights an importance of platelet α_IIb_β_3_ phosphorylation and outside-in signaling for the formation of a stable platelet thrombus as well as for microparticle release by platelets. These two phosphorylation sites (Y747 and Y759) within the β_3_ integrin cytoplasmic domain regulate arterial thrombosis under high sheer, without affecting hemostasis [9]. Therefore, targeting of these sequences within the β_3_ integrin might represent an attractive therapeutic strategy with a potential of developing drugs diminishing arterial thrombosis without causing serious bleeding complications, which is one of the main drawbacks of existing anti-platelet strategies. This has major implications for various pathologies associated with platelet hyperreactivity, such as arterial thrombosis leading to myocardial infarction and, especially, stroke, as well as thrombotic complications in cancer patients, where excessive bleeding is associated with a high risk of morbidity. Thus, disruption of integrin function by targeting the β_3_ phosphorylation motifs could provide new and potentially safer therapeutic interventions for numerous thrombotic complications.

## Conclusion

This study shows that the phosphorylation status of α_IIb_β_3_ on platelets, but not α_V_β_3_ on leukocytes or endothelial cells, is critical for the formation of a stable thrombus. Therefore, β_3_ phosphorylation and the resulting outside-in α_IIb_β_3_ signaling is a primary mechanism regulating not only the consolidation and stabilization of platelet aggregation [13] but also platelet procoagulant activity.

## Abbreviations

ADP: Adenosine 5′-diphosphate
DiYF: knock-in mouse strain, where two β_3_ cytoplasmic tyrosines are mutated to phenylalanine
PBS: phosphate-buffered saline
PMA: Phorbol-12-myristate-13-acetate
PRP: Platelet rich plasma
WT: Wild type
